# Speeding Up Microevolution: The Effects of Increasing Temperature on Selection and Genetic Variance in a Wild Bird Population

**DOI:** 10.1371/journal.pbio.1000585

**Published:** 2011-02-01

**Authors:** Arild Husby, Marcel E. Visser, Loeske E. B. Kruuk

**Affiliations:** 1 Institute of Evolutionary Biology, School of Biological Sciences, University of Edinburgh, Edinburgh, United Kingdom; 2 Department of Animal Ecology, Evolutionary Biology Centre (EBC), Uppsala University, Uppsala, Sweden; 3 Netherlands Institute of Ecology (NIOO-KNAW), Heteren, The Netherlands; The University of North Carolina, United States of America

## Abstract

The amount of genetic variance underlying a phenotypic trait and the strength of selection acting on that trait are two key parameters that determine any evolutionary response to selection. Despite substantial evidence that, in natural populations, both parameters may vary across environmental conditions, very little is known about the extent to which they may covary in response to environmental heterogeneity. Here we show that, in a wild population of great tits (*Parus major*), the strength of the directional selection gradients on timing of breeding increased with increasing spring temperatures, and that genotype-by-environment interactions also predicted an increase in additive genetic variance, and heritability, of timing of breeding with increasing spring temperature. Consequently, we therefore tested for an association between the annual selection gradients and levels of additive genetic variance expressed each year; this association was positive, but non-significant. However, there was a significant positive association between the annual selection differentials and the corresponding heritability. Such associations could potentially speed up the rate of micro-evolution and offer a largely ignored mechanism by which natural populations may adapt to environmental changes.

## Abstract 2

The authors show that environmental variation may lead to a positive association between the annual strength of selection and expression of genetic variance in a wild bird population, which can speed up microevolution and have important consequences for how fast natural populations adapt to environmental changes.

## Author Summary

The speed of evolutionary change in a phenotypic trait is determined by two key components: the amount of genetic variance underlying the trait and the strength of selection acting on it. Many studies have shown that both selection and expression of genetic variance may depend on the environmental conditions the population experiences. However, the possibility that the strength of selection and the expression of genetic variance become positively or negatively associated as a result of this environmental covariance, so as to speed up or hamper an evolutionary response, has been largely ignored. Here we show that, in a wild bird population, the annual strength of selection on and the expression of genetic variance in timing of breeding (a key life history trait) are positively associated due to changing environmental conditions (warmer temperatures). Such a positive association should potentially speed up any microevolutionary response to selection (such as that imposed by climate warming). Our results illustrate the existence of substantial temporal variation in response to environmental heterogeneity, and thus highlight a so far neglected mechanism that may be important in determining the evolutionary dynamics in natural populations.

## Introduction

Predicting an evolutionary response to selection in a phenotypic trait requires knowledge of the strength of selection acting on the trait and its genetic basis. Although it has long been recognized that the strength, and direction, of selection may vary with environmental conditions (e.g., [1]), widespread recognition of the fact that additive genetic variance (and thus heritability) may also change with environmental conditions has been more recent [2],[3]. Taken together, these observations generate an expectation of an environmentally driven association between the two parameters that, in theory, has the potential to either enhance (positive association) or constrain (negative association) any response to selection. Surprisingly, however, to our knowledge only one study to date has quantified the association between annual estimates of selection and expression of genetic variance (measured as heritability) in a heterogeneous environment [4]. In this article, we present data from a long-term study of a great tit (*Parus major*) population known to be experiencing substantial shifts in climatic conditions, and test for the effects of the novel environmental conditions on the expression of additive genetic variance, and the selection on, a key life history trait, breeding time.

Many studies have found that selection is often strongest when environmental conditions are adverse (e.g., [4]–[8]), and there is a clear indication that “perturbed or stressed” populations have larger standardized selection differentials than “undisturbed” populations ([9] p. 208). For example, Garant and co-workers [5] examined selection on fledgling body mass in a population of great tits and found that selection differentials were greater in years when average body mass was low and when the proportion of individuals surviving to recruitment was low, both indicative of poor/adverse environmental conditions. In general, therefore, selection is often stronger when environmental conditions are adverse.

Unlike the general tendency for selection to be stronger in adverse environments, conclusions regarding the effects of good versus adverse environments on the expression of additive genetic variance are more mixed. Laboratory studies investigating the effect of environmental conditions have generally found a weak tendency for heritability to increase in stressful environments with this being caused by changes in both the expression of genetic variance as well as the environmental variance (reviewed in [10]). This pattern, however, is in contrast to most studies from natural populations that find, at least for morphological traits, that additive genetic variance and heritability is often relatively lower in unfavorable conditions [3],[10],[11].

It is important to realize that heritability (h^2^) may change under different environmental conditions either because of changes in additive genetic variance (V_A_) or other variance components (e.g., permanent environmental variance (V_PE_) or residual variance (V_R_)). However, changes in V_A_ are of particular interest because they indicate a change in the “evolvability” [12], or the potential to respond to selection, of a trait. Furthermore, changes in V_A_ can only be due to a change in the genetic architecture of a trait through mechanisms such as genotype-environment interactions, changes in mutation and recombination rates, and removal of alleles with low fitness by selection (reviewed in [10]). Depending on the direction and scale of these changes, both additive genetic variance and heritability may increase or decrease depending on the relative impact of each of the above factors [10].

The possibility that both the expression of additive genetic variance of a trait and the strength of selection acting on it may vary with environmental conditions is significant, as such environmentally induced variation may be important in determining the evolutionary dynamics of natural populations. In particular, the observation of a general increase in genetic variance of morphological traits [3],[10],[11] and a reduction in selection [4],[6] during favorable conditions in natural populations leads to the expectation of a negative relationship between genetic variance and the strength of selection, such that selection should be strongest in years in which the expression of additive genetic variance is least. This association could severely constrain a response to selection and provide one explanation for the frequently observed scenario of apparent stasis in natural populations [4]. However, in contrast to morphometric traits, life history traits do not appear to show a clear indication of increased heritability in stressful environments [3]. This makes it more difficult to predict how, or if, additive genetic variance and selection on life history traits may covary in a heterogeneous environment.

Surprisingly, despite the potential importance of environmentally induced associations between the strength of selection and expression of genetic variance, we are aware of only one previous study that has tested for such an association. Wilson et al. [4] found that the strength of selection on body weight in a free-living population of Soay sheep (*Ovis aries*) in a given year was negatively correlated with the expression of total genetic variance (assessed via the heritability) of body weight, suggesting a possible constraint on the potential for evolution of body weight in this species. However, so far no study has, to our knowledge, examined the association between strength of selection and V_A_ (or h^2^) in a life history trait. Hence, we do not know if such relationships are common in nature, and whether they are generally negative, which may constrain an evolutionary response, or whether there are examples of positive associations between strength of selection and V_A_ (or h^2^), which would speed up an evolutionary response.

Here we use data from an exceptionally long-term study population of great tits (*Parus major*) in the Hoge Veluwe, the Netherlands, to investigate how selection and expression of additive genetic variance of a key life-history trait (timing of breeding, or “laying date”) vary in relation to rapid changes in environmental conditions (spring temperature). The evolutionary response in a trait between generations can be predicted as R  =  V_A_ * β [13],[14], where β is the selection gradient, defined as the covariance between relative fitness and trait value divided by the phenotypic variance in the trait (i.e., β  =  cov(ω,trait)/V_Ptrait_) [15]; we therefore test the association between V_A_ and the selection gradients β under different environmental conditions. We also consider the alternative format for predicted response, R  =  h^2^ * S [16], where S is the selection differential, defined as the covariance between relative fitness and trait value (i.e., S  =  cov(ω,trait)) and test for an association between heritability and selection differentials.

This system is particularly well suited to an exploration of the association between selection and V_A_ in a variable environment because phenotypic data, pedigree data, and a thorough understanding of how environmental conditions influence laying date are available [17],. Previous studies in this population have reported a significant increase in spring temperature over the past four decades [18] and have also shown that warm spring temperatures lead to earlier laying dates [17]. Furthermore, warmer temperatures lead to reproduction being mistimed relative to the food peak [17], resulting in a decrease in both the number and size of fledglings [19], and in the proportion of females producing a second clutch [20]. Spring temperatures are thus not only directly related to observed variation in laying dates but can also be used as a measure of environmental quality in the population. In addition, spring temperatures are now significantly above those which the population has previously experienced [18], providing an ideal opportunity to study how novel environmental conditions may influence evolutionary dynamics. We therefore tested the temperature dependence of the selection gradients and differentials, how expression of additive genetic variance and heritability changed with temperature, and finally, how the measures of selection were associated with the amount of genetic variance present in the population.

## Results

### Environmental Dependence of Strength of Selection

**Figure 1:**
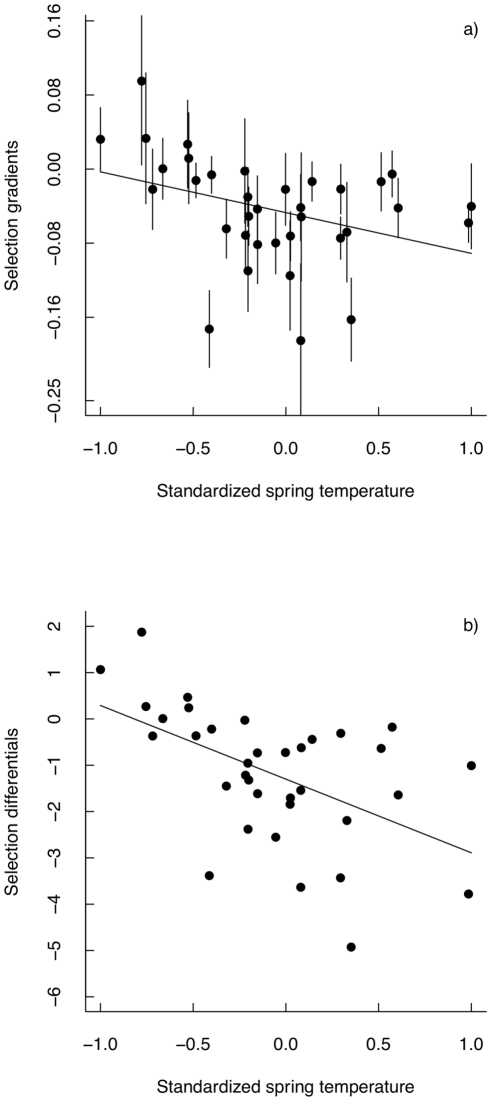
Environmental dependent strength of selection. Annual selection gradients and selection differentials on laying date regressed against standardized spring temperature. Each point is the estimated temperature-specific selection gradient (β) and selection differential (S) in (a) and (b), respectively. The solid line represents the least squares regression lines of selection gradients (Figure 1a) and selection differentials (Figure 1b) on spring temperature.

We found, firstly, strong selection on laying date, with early breeding birds having higher fitness than late breeding individuals (Table 1). Indeed, 29 out of the 35 estimates of annual selection gradients and differentials were negative (Figure 1, Table S2), reflecting general selection for earlier breeding, as has previously been shown in this population [17],[21]. Secondly, the interaction between laying date and standardized spring temperature was significantly negative (Table 1), indicating that with increasing spring temperatures the relationship (slope) between fitness and laying date became more negative (i.e., slope steeper in warmer years). Consequently, selection for early breeding was significantly stronger (indicated by more negative values of β) in warm years than in cold years; i.e., the strength of selection on lay date varied with environmental conditions (Figure 1). This result was confirmed by regressing the annual selection gradients (β) against temperature: there was a significant increase in the (absolute) magnitude of the strength of selection with increasing temperatures (regression slope  = −0.044, se  = 0.019, t_33_  = −2.203, *p* = 0.035, Figure 1a). The results were the same for selection differentials (regression slope  = −1.589, se  = 0.450, t_33_  = −3.529, *p* = 0.001, Figure 1b).

**Table 1:** Mixed model selection analysis of effects of laying date (LD) and mean-centered spring temperatures (TEMP) on the number of offspring recruited to the breeding population each year.

Effect	b ± SE	Wald Statistics	*p* Value	Variance (SE)
**Random: Individual identity**				0.295 (0.044)
Year				0.638 (0.168)
**Fixed: LD**	−0.041±0.006	50.556	<0.001	
TEMP	0.151±0.202	1.515	0.218	
LD × TEMP	−0.015±0.006	6.017	0.014	

Analysis is based on a total of 3,852 records from 2,394 different individuals over a 35-year period (1973–2007). The models were fitted in ASREML-R using a Poisson error structure (log link function) with individual identity and year included as random effects. Significance of fixed effects was assessed based on their Wald test statistics, distributed as χ^2^ each with 1 d.f.

### Environmental Dependence of Additive Genetic Variance and Heritability

Comparing a model in which the additive genetic and permanent environment components of variance (V_A_ and V_PE_) in a given year were constant across different spring temperatures to one in which V_A_ and V_PE_ could vary with the temperature gave strong support for environmental dependence of V_A_ and V_PE_ (χ^2^_4_ = 74.90, *p*<0.001). Consequently, we used the predictions from the model in which the two variance components varied with spring temperature to generate estimates of annual V_A_ and h^2^ and to explore how these annual estimates corresponded to the observed changes in the strength of selection on laying date.

**Figure 2:**
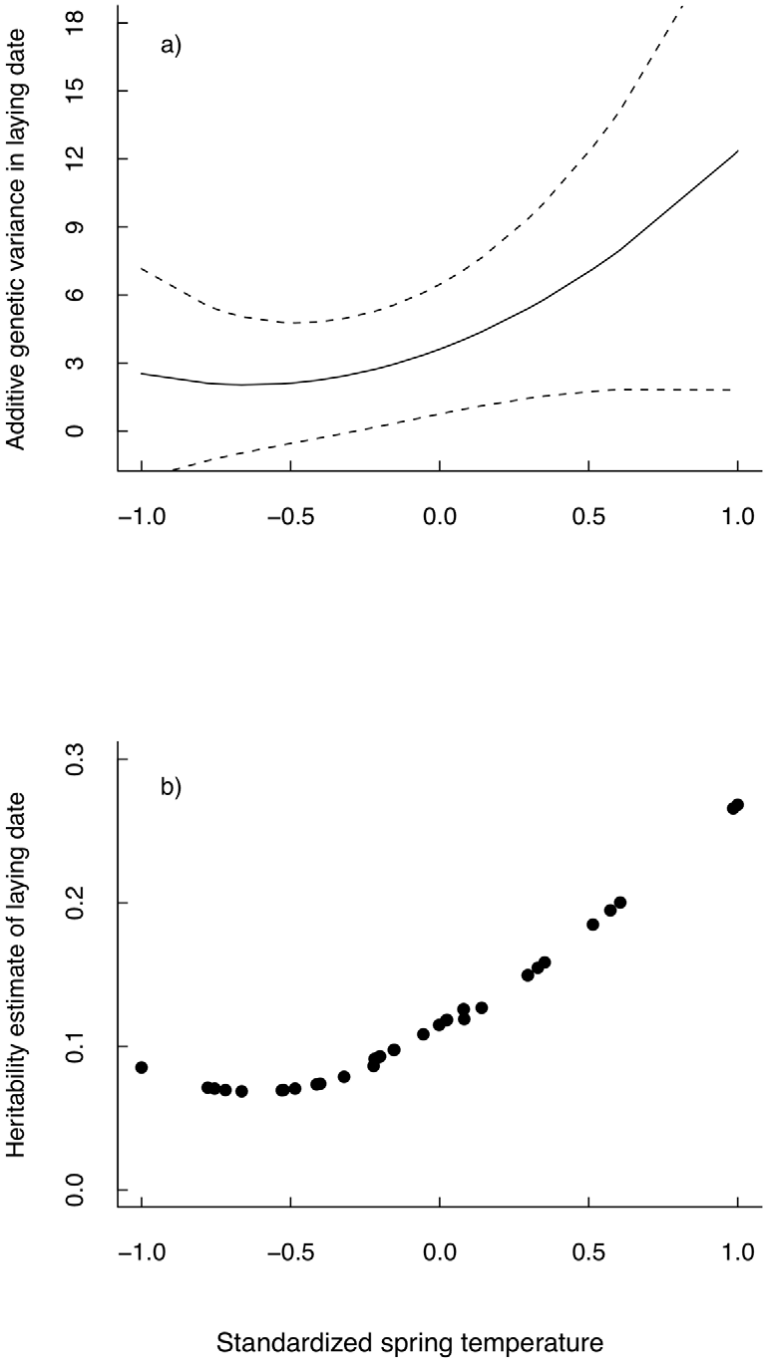
Changes in V_A_ and h^2^ with spring temperature. (a) Estimated change in additive genetic variance (V_A_) with 95% confidence interval against standardized spring temperature as predicted from the random regression animal model in which V_A_ and V_PE_ vary with temperature. (b) Estimated change in heritability across spring temperature as predicted from a model where V_A_ and V_PE_ changed with standardized spring temperature; each point represents the year specific (and thus temperature specific) h^2^ estimate.

The estimated environment-specific **G**-matrix predicted a substantial increase in V_A_ with increasing standardized spring temperatures (Figure 2a, each point represents an environment-specific V_A_ estimate). Similarly, there was a corresponding increase in the year-specific heritability estimates with increasing temperature (Figure 2b, each point represents a environment-specific h^2^ estimate).

### Association Between Strength of Selection and Additive Genetic Variance

We then tested whether the effects of increasing temperature on selection and genetic variance generated an association between them.

**Figure 3:**
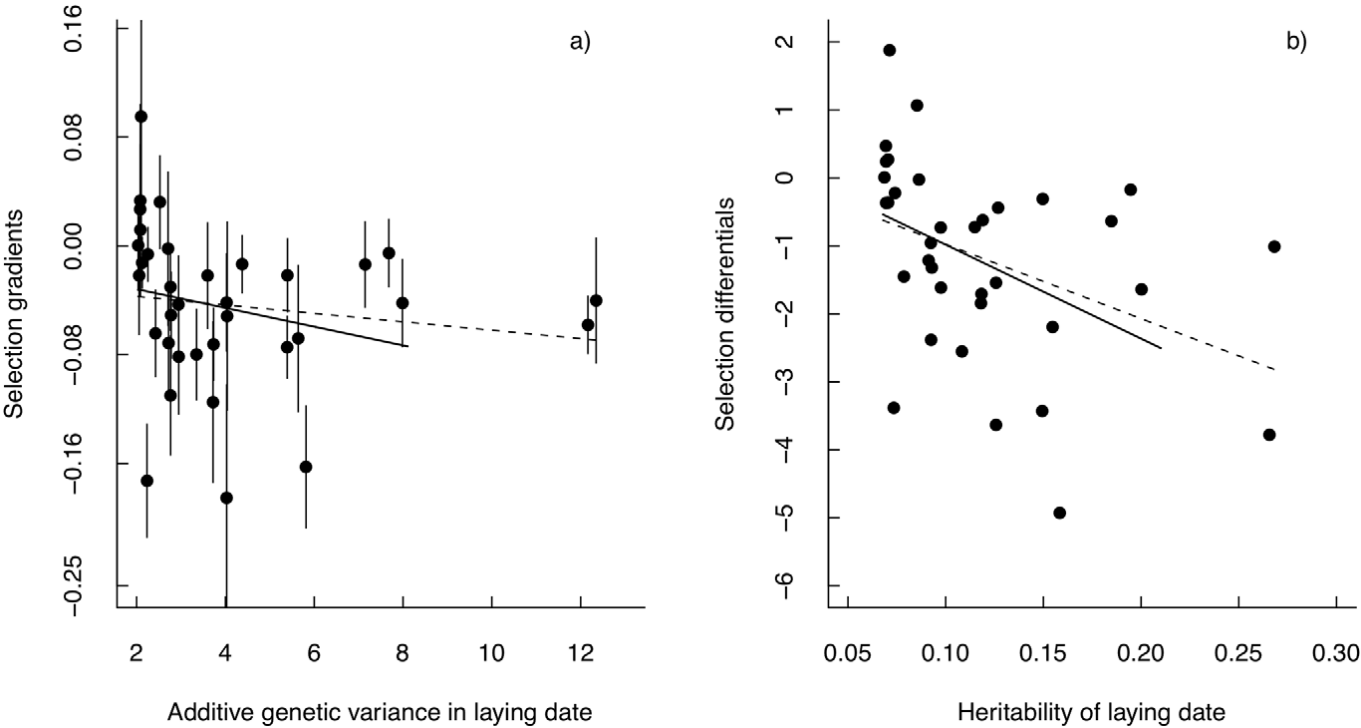
Relationship between selection and V_A_ and h^2^. (a) Annual selection gradients against annual estimated V_A_ with the estimated regression line using all data (dotted line) and data where V_A_ outliers were removed (solid line); see text for further details. (b) Annual selection differentials against annual estimated h^2^ with the estimated regression lines from a least squares regression using all data (dotted line) and data where h^2^ outliers were removed (solid line); see text for details.

The relationship between the selection gradients (β) and additive genetic variance (V_A_) for laying date was negative but non-significant (slope = −0.006, se = 0.005, t_33_ = 1.18, *p* = 0.25; Figure 3a, dotted line). However, as random regression models are known to give upwardly biased estimates at the endpoints of the polynomials [22], we also tested this relationship after removing the extreme V_A_ outliers (V_A_ >10, see Figure 3a). This resulted in a near-significant relationship between the two (slope = −0.014, se = 0.008, t_31_ = 1.84, *p* = 0.075; Figure 3a, solid line). Furthermore, there was a significant negative relationship between the selection differentials S and heritability (slope = −10.96, se = 4.43, t_33_ = 2.48, *p* = 0.019, Figure 3b), which was robust to excluding the two extreme heritability estimates (excluding h^2^ >0.25: slope = −13.82, se = 6.2, t_31_ = 2.23, *p* = 0.03). Finally, using standardized measures of selection, there was a negative although non-significant significant relationship between selection and additive genetic variance and a significantly negative relationship between strength of selection and heritability (see Text S1).

**Figure 4:**
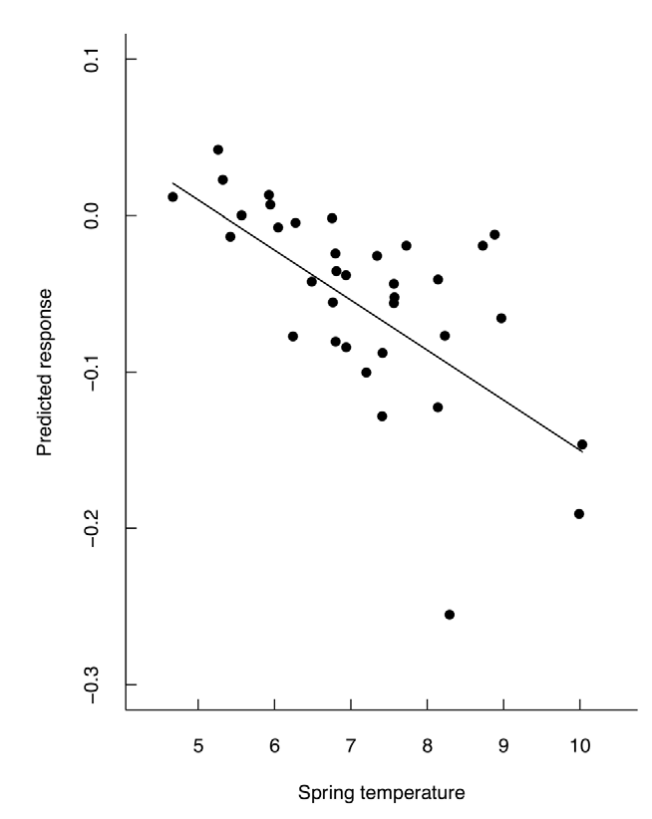
Environmental dependent response to selection. The environmental covariance between strength of selection (measured as the selection gradient) and expression of additive genetic variance as a function of the environment leads to a strong relationship between the predicted response to selection (measured using a modified version of Lande's equation, see Text S1) and temperature (least squares regression: slope = −0.031, se = 0.006, t_33_ = −4.92, *p*<0.001).

Note that because there is selection for early breeding, selection gradients and differentials are negative, but there is a *positive* association between the absolute *strength* of selection and levels of additive genetic variance (or heritability). As a result, in years in which selection on laying date was relatively strong, estimated V_A_ (and h^2^) was higher than in years when selection was weak (Figure 3). This association resulted in a highly significant relationship between temperature and the magnitude of the predicted response to selection (Figure 4).

## Discussion

Our analysis of long-term records on an important life history trait in a wild bird population found evidence that in years when spring temperatures were highest, selection was strongest, and the magnitude of estimates of additive genetic variance V_A_ (and hence heritability) was also highest. As a result, there was evidence of a positive association between the strength of selection and the expression of additive genetic variance, and heritability. A positive association such as this between the strength of selection and expression of genetic variance and heritability could make the magnitude of the response strongly environmentally dependent; in this case, warming temperatures would considerably enhance any expected response to selection.

As has generally been found in studies of selection on laying date in birds [17],[21],[23],[24],[25], selection gradients and differentials were generally negative, indicating that early-breeding individuals had higher fitness than late-breeding individuals. Furthermore, the strength of selection was strongest when temperatures were highest (Figure 1). It has previously been shown that reproductive success [26] has declined in this population over the study period, most likely because, with increasing spring temperatures, there is evidence of increased “mistiming” of reproduction relative to the peak in food abundance [17]. This decline in reproductive success suggests that high spring temperature is generally associated with adverse environmental conditions. Hence, our results confirm the expectation in natural populations of stronger selection in adverse environmental conditions [9]. It is important to point out, however, that high temperatures are not necessarily associated with adverse environmental conditions in other systems. For example, a population of great tits in the U.K. has also experienced increasing temperatures, but recruitment rates in this population have increased over time [27].

Previous studies on natural populations have found that heritability decreased when environmental conditions are stressful [3],[10], although we know less about how V_A_ changes. Here, we found instead that both additive genetic variance and heritability of laying date increased rather than decreased (Figure 2). Although there was substantial evidence that V_A_ and V_PE_ changed with environmental conditions (see Results), the change in V_A_ alone was not statistically significant [18], something that is reflected in the large standard errors in Figure 2a. However, the statistical power to detect significant changes in additive genetic variance in relation to varying environmental conditions using a random regression animal model approach may be limited [18],[28]. Most importantly, the increase in V_A_ is very large and represents 81.4% of the total change in V_P_ (Figure 2a). This increase in V_A_ is, for example, much larger than the increase in maternal genetic variance (V_M_) for birth weight in Soay sheep [4]. Note also that in the Soay sheep analysis, maternal environmental effects were not fitted with the same order polynomials as the maternal genetic effects, so that some of the increase in maternal genetic effects variance estimates could potentially be driven by environmental rather than genetic effects (in the same way as permanent environment variance will inflate additive genetic variance if not fitted explicitly, [29]).

One possible explanation for why V_A_ may increase with higher temperatures is that high temperatures constitute not only a stressful, but also a novel, environment. For example, 2005 and 2007 had the highest recorded spring temperatures since this population study began back in 1955. It has been suggested that V_A_ could increase in novel environments because selection has not yet had the possibility to remove the most deleterious alleles, as it will have in the ancestral environment, thereby causing an increase in the standing genetic variation [30]; a suggestion that has been confirmed in some empirical studies [31],[32]. More generally, our finding adds support to the idea [3] that predicting the direction in which V_A_ should change with environmental conditions is complicated when environmental changes also leads to novel conditions, as is often the case with human-induced changes [3].

The increase in V_A,_ heritability and strength of selection with increasing spring temperature meant that there was a positive association between the strength of selection on laying date and the heritability as well as expression of additive genetic variance of laying date (Figure 3a and b, respectively). The relationship between selection and amount of genetic variance was in the same direction whether using β as the measure of selection and V_A_ as the measure of the potential for the population to adapt, versus using S and h^2^, but it was stronger (and hence statistically significant) between S and h^2^ (Figure 3b) than between β and V_A_ (Figure 3a). One possible explanation for this may be that in the S and h^2^ comparison, both parameters depend on V_P_ whereas in the β and V_A_ comparison only β depends on V_P_ and thus a change in V_P_ may more quickly lead to a disassociation between β and V_A_ than between S and h^2^.

Nevertheless, we believe the fact that the relationships between β and V_A_ and between S and h^2^ are in the same direction (as well as that between standardized selection and V_A_/h^2^; see Text S1) offers strong support for an environmental coupling between these two parameters. This conclusion is supported by a highly significant temperature dependence of the predicted response to selection (see below, Figure 4).

Following traditional methodology we predicted the expected response to selection (see Text S1) using the Lande equation: R  =  V_A_ * β [13],[14] but correcting for overlapping generations and the sex-limited expression of laying date, with the year-specific V_A_ and β estimates (see Table S2), which amounted to an advance of 1.81 days in total over the study period. Furthermore, using the average of the annual V_A_ and β values gave a predicted response of 1.46 days advancement, which corresponds to only 81.1% of the predicted response using year-specific values. Thus, not incorporating environmental dependence of the expression of genetic variance and strength of selection may underestimate the predicted response by up to 20%, at least in this specific case. Failing to incorporate an environmentally dependent association between the strength of selection and genetic variance may further obscure our understanding of microevolution as the predicted response will be dependent on the environmental variable in question. For example, in our study the predicted response is strongly correlated with spring temperature, with a much larger predicted response in warmer temperatures compared to cold (Figure 4). We caution, however, that the breeder's equation (and equivalent Lande equation) has particularly poor success when applied to studies in natural populations [33], presumably because many of its underlying assumptions are not met (see Text S1 for further discussion on this topic).

Very few studies have simultaneously examined how environmental factors influence genetic expression and selection and the association between them. Indeed, we are only aware of this being examined in a Soay sheep population [4], where there was a negative association between the strength of selection and heritability of body size. Another example where there may be a negative association between the strength of selection and heritability is for juvenile growth rates in North American red squirrels (*Tamiasciurus hudsonicus*) [34]. Although this study did not explicitly consider the association between selection and genetic variance, they found that V_A_ and maternal genetic variance increased in years with low cone abundance (poor environment) whereas viability selection was stronger in years of high cone abundance (due to competition for territories [34]). This should generate a negative association between selection and total genetic variance that may hamper a response to selection.

Our results thus demonstrate a relatively unexplored mechanism that could potentially increase the speed of adaptation to climate change in this population. As temperatures are expected to continue to increase [35], a positive association between strength of selection on laying date and its potential to evolve may prove an important factor allowing at least this specific population to adapt to the rapid environmental conditions experienced. As it is ultimately this rate of adaptation that is crucial if species are to cope with climate change [36], our findings suggest that models linking population viability to climate change should incorporate such dynamic processes.

## Materials and Methods

### Study System and Data Collection

The data were collected in the Hoge Veluwe National Park, the Netherlands (52°05′ N, 05°50′ E), during the period 1973 to 2007. Nest boxes were visited at least once every week during the breeding season (April–June). The laying date of the first egg of a female's clutch (laying date, LD) was calculated from the number of eggs found during the weekly checks, assuming that one egg was laid per day. Both parents were caught and individually marked on the nest using a spring trap when the young were 7–10 d old. Laying dates are presented as the number of days after March 31 (day 1 =  April 1, day 31 =  May 1). We only used information on the first clutch, defined as any clutch started within 30 d of the first laid egg in any given year. Replacement and second clutches (which currently compromise <5% of breeding attempts, 21]) were thus excluded from the analysis. In total, therefore, we had information about 3,852 breeding records from 2,394 females. More details about the study population can be found in van Balen [37].

Temperature data were obtained from the De Bilt weather station of the Royal Dutch Meteorological Institute (www.knmi.nl/klimatologie/daggegevens) and used to calculate the daily average temperature over the period March 13–April 20, which is the period that best predicts the onset of laying using a sliding window analysis (see [18] for more detail).

### Selection Analysis

To test for a relationship between spring temperature and the strength of selection on laying date, we took two approaches. First, we used a generalized linear mixed effects model (GLMM) with a Poisson error link fitted in ASREML-R [38] to model the relationship between number of recruits a female produced for the given year (as the measure of fitness) and her laying date that year, and to test its dependence on spring temperature (as measured by the interaction term between laying date and spring temperature). Individual identity and year were included as random effects to account for repeated measures on the same individuals and on years. Second, we estimated the annual strength of selection using the number of recruits produced per year divided by the mean number of recruits produced in the given year as a measure of relative fitness (ω) for each individual. Selection was then measured as the selection gradients (β) defined as the covariance between relative fitness and observed laying date divided by the variance in observed laying date, i.e. β  =  cov(ω, LD)/V_PLD_. Using this measure of selection allows us to predict the response to selection using the Lande equation: R  =  V_A_*β [13]. Predicting the response to selection can also be done using the more familiar Breeder's equation, R  =  h^2^*S [16], which uses an alternative measure of selection, the selection differentials defined as the covariance between a female's relative fitness (ω) and her observed laying date (LD), i.e. S  =  cov(ω, LD) [15]. Because a previous study examining the association between strength of selection and expression of genetic variance used S and h^2^ as parameters [4], we also present our results using these parameters for comparison. We note, however, that using selection gradients may represent a better measure of selection when the phenotypic variance in a trait changes [14], which it does here. We then regressed the annual selection gradients (and differentials) against the environmental values using a least-squares regression (with 1/se^2^ as weights when considering the selection gradients) in R 2.8.0 [39].

Finally, to allow comparison with other studies [40], we repeated all selection analyses using variance-standardized laying dates (i.e. standardizing laying date values to have zero mean and unit variance within each year). This did not change our conclusions and we report the results from these analyses in the Supporting Information section (Text S1, Table S1).

Yearly spring temperature values, standardized spring temperature values, sample size, mean laying dates, selection gradients (β), selection differentials (S), standardized selection differentials, estimated additive genetic variance, and heritability estimates along with annual predicted responses to selection (V_A_*β) are all reported in Table S2.

### Pedigree Structure

Quantitative genetic analyses require knowledge about the relationships among individuals within a population. Here, a pedigree was constructed where all ringed females known to have bred were assigned a mother and father as determined from observational data. In cases where brood manipulation experiments had been carried out and chicks had been moved between nests, we assigned the genetic parent rather than the social parent. If only one parent was known, we “dummy coded” the missing parent to preserve sibship information (note that we did not assign a phenotype to this parent). The extra-pair paternity (EPP) rate is unknown in this population, but is generally found to be low (3%–9%) in other populations of great tits [41],[42] and as extra pair paternity rates of less than 20% have been shown to have a negligible impact on heritability estimates [43] using a social pedigree is unlikely to be problematic.

### Quantitative Genetic Analyses

Phenotypic trait variances can be separated into genetic and environmental causes of variation using an “animal model” [44]–[46]. By maximizing the information available in an extensive multi-generational pedigree, the “animal model” minimizes upward inflation of estimates of additive genetic variance (V_A_) due to shared environmental effects between relatives; this approach has been shown in simulation studies to perform well in partitioning environmental and genetic components of variance [29]. There are several additional reasons to believe that the genetic and environmental components have been well separated here. First, a previous study found no indication that common environment effects in the form of maternal effects are important for laying date in this population (V_M_/V_P_  = 0.0023 [47]). Second, although common environmental effects frequently play a major role in inflating covariances between relatives in nestling traits [48], this is rarely the case for traits that are only expressed as adults, like laying date which we study here. Third, we explicitly take common environmental effects into account by fitting a permanent environmental effect [45]. In summary, therefore, we believe that our estimates of V_A_ and h^2^ are accurate and unbiased by inflation of common environment effects.

Rather than only estimating the amount of genetic and environmental variance in laying date, we are interested here in whether the variance components changed with environmental conditions, and we therefore used a “random regression animal model” [49]. Random regression models use covariance functions [50] to explicitly fit variance components as a function of the environment and hence allow a detailed examination of how environmental heterogeneity—in this case, spring temperature—influences genetic architecture. Thus our model was:
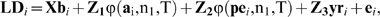
(1)where **LD** *_i_* is the vector of the individual (*i*) laying dates and **X, Z_1_, Z_2_**, and **Z_3_** are the design and incidence matrices relating to the fixed and random effects of the additive genetic (**a**_i_), permanent environment (**pe**_i_), and year (**yr**_i_) observations, respectively. T is the spring temperature each year standardized to a (−1, 1) interval (Table S2). Fixed effects (**b** ***_i_*** vector) included age as a two-level factor (first year breeder or older), to correct for the fact that laying date is generally later in young birds compared to older birds in great tits [51], and spring temperature to account for the population-level response in mean trait value. Year (**yr** vector) was included as a random effect in order to model variation between years not explained by spring temperature and a permanent environment effect (**pe** *_i_* vector) was fitted because of the repeated sampling of the same individuals; this also reduces inflation of estimates of the additive genetic variance due to environmental factors [29]. The error term (**e** vector) was partitioned into three decade–specific (1973–1984, 1985–1996, 1997–2007) groups, thus allowing residual errors to vary between decades. ϕ(**a**_i_,n_1_,T) is the random regression function of order n_1_ of the additive genetic effect of individual *i*, which varies as a function of the temperature T in a given year, and similarly, ϕ(**pe** *_i_*,n_2_,T), is the random regression function of order n_2_ of the permanent environment effect varying as a function of T.

Because we were only interested in whether the two variance components (and particularly V_A_) changed with the environment, we only fitted two models. The first model was a zero order function (n_1_ = n_2_ = 0) for both V_A_ and V_PE_ in which variance components are constant across the environment. In the second model, we fitted a first order polynomial (n_1_ = n_2_ = 1) for both V_A_ and V_PE_, thus allowing both additive genetic effects and permanent environment effects, and hence their corresponding variance components, to vary across the environment T. These two models were then compared using a likelihood-ratio test by calculating twice the difference in log likelihood, which is chi-squared distributed with degrees of freedom equal to the difference in degrees of freedom between the two models [52], which is here equal to 4 (variance in slopes and covariance between elevation and slope for both V_A_ and V_PE_). As the model where both variance components were allowed to vary was significantly better than a model in which they were assumed to be constant (see Results) we used the estimates from the first order polynomial model to generate predictions of annual values of V_A_ (and V_PE_) across varying temperatures. The environment-specific additive genetic covariance matrix, **G**, was then obtained as **G**  =  **zQz** ^T^, where **z** is the vector of orthogonal polynomials evaluated at standardized temperature values and **Q** is the additive genetic variance-covariance matrix of the random regression parameters. Approximate standard errors for the (co)variance components of **G** as a function of the temperature values were calculated according to Fischer et al. [22], with confidence intervals defined as twice the standard errors. Finally, environment-specific heritability estimates were calculated as the environment-specific V_A_ estimate divided by the environment-specific V_P_ estimate from the model in which both V_A_ and V_PE_ varied with the environment. Because it has been found that random regression models can be sensitive to “edge effects” [22],[53], we repeated our analyses where we look at the association between strength of selection and expression of genetic variance to be conservative. For more information about the use of random regression animal models in natural populations, see [18] and [54]. All animal models were fitted using REML methods implemented in ASReml v 2.0 [38].

### Association between Strength of Selection and Expression of Genetic Variance

In order to test for an association between the strength of selection operating on laying date and the expression of additive genetic variance in laying date, we used environment-specific (and thus annual) V_A_ and h^2^ estimates generated from the random regression animal model and regressed the annual selection gradients on our annual estimates of V_A_; we then repeated the regression for annual selection differentials against h^2^. Regressions using selection gradients were weighted by the inverse of the square of the standard error.

Because some individuals bred in multiple environments (i.e. years), estimates of selection will not be entirely independent, potentially violating some of the assumptions of least squares regression analyses. Although this is an inherent problem to all longitudinal studies, we assessed the potential for it to bias our conclusions by repeating our selection analyses using only a single record per individual (its first breeding attempt). Because this did not change the direction or significance of our analyses (regression of β on V_A_ using 1/se^2^ as weights: b = −0.009, se = 0.005, t_33_ = −1.70, *p* = 0.099; regression of S on h^2^: b = −14.54, se = 5.08, t_33_ = −2.86, *p* = 0.007), we conclude that the potential violation of the non-independence criteria caused by multiple breeding events from the same individuals is not a significant issue here.

Although annual estimates of V_A_ and h^2^ are derived from the random regression model, note that in testing for a relationship between them and selection, we use them only as predictor variables in a regression, for which there need not be an assumption of independence of data points.

## Supporting Information

Table S1 Environmentally dependent strength of standardized selection on laying date.
(0.03 MB DOC)

Table S2 Yearly sample size, selection estimates, quantitative genetic parameters, and predicted response to selection.
(0.09 MB DOC)

Text S1 Environmentally dependent strength of standardized laying date selection and the covariance between standardized laying date selection with additive genetic variance and heritability.
(0.03 MB DOC)
