# Supplementary material for: Speeding Up Microevolution: The Effects of Increasing Temperature on Selection and Genetic Variance in a Wild Bird Population
Source: PLoS Biol. 2011 Feb 1;9(2):e1000585. doi: 10.1371/journal.pbio.1000585 (PMC3051266; doi:10.1371/journal.pbio.1000585)
Supplement: Table S1 — Environmentally dependent strength of standardized selection on laying date. (0.03 MB DOC) [file pbio.1000585.s001.doc]

**Table S1.**

Mixed model selection analysis of effects of standardized laying date (sLD) and mean-centred spring temperatures (TEMP) on the number of offspring recruited to the breeding population each year. Analysis is based on a total of 3852 records from 2394 different individuals over a 35 year period (1973-2007). The models were fitted in ASREML-R using a Poisson error structure (log link function) with individual identity and year included as random effects. Significance of fixed effects was assessed based on their Wald test statistics, distributed as χ^2^ each with 1 d.f.

| Effect | β ± SE | Wald  statistics | *P* - value | Variance (SE) |
| --- | --- | --- | --- | --- |
| Random: Individual identity |  |  |  | 0.295 (0.044) |
| Year |  |  |  | 0.636 (0.167) |
| Fixed: sLD | -0.215 ± 0.030 | 47.424 | < 0.001 |  |
| TEMP | -0.018 ± 0.147 | 0.001 | 0.987 |  |
| sLD X TEMP | -0.109 ± 0.034 | 10.488 | 0.001 |  |
