# Supplementary material for: Speeding Up Microevolution: The Effects of Increasing Temperature on Selection and Genetic Variance in a Wild Bird Population
Source: PLoS Biol. 2011 Feb 1;9(2):e1000585. doi: 10.1371/journal.pbio.1000585 (PMC3051266; doi:10.1371/journal.pbio.1000585)
Supplement: Table S2 — Yearly sample size, selection estimates, quantitative genetic parameters, and predicted response to selection. (0.09 MB DOC) [file pbio.1000585.s002.doc]

**Table S2:** Yearly spring temperature, standardized spring temperature, sample size (*n* = number of recruits), unstandardized selection differentials, unstandardized selection gradients, standard error of the selection gradients, additive genetic variance, approximate standard errors of the additive genetic variance and heritability estimates for laying date in great tits as well as the predicted response to selection (see Text S1 for details on how this was calculated).

| Year | Spring temp. | Standardized spring temp. | *n* | Mean laying date | Selection differentials (S) | Selection gradients (β) | SE of selection gradients | Standardized selection differentials | SE of standardized selection differentials | Additive genetic variance (V_A_) | SE of additive genetic variance (V_A_) | Heritability Estimates | Predicted response |
| --- | --- | --- | --- | --- | --- | --- | --- | --- | --- | --- | --- | --- | --- |
| 1973 | 5.422 | -0.719 | 43 | 28.317 | -0.367 | -0.022 | 0.043 | -0.089 | 0.178 | 2.057 | 1.593 | 0.070 | -0.014 |
| 1974 | 8.885 | 0.573 | 77 | 16.343 | -0.176 | -0.005 | 0.025 | -0.031 | 0.143 | 7.679 | 2.938 | 0.195 | -0.012 |
| 1975 | 4.668 | -1.000 | 31 | 33.393 | 1.066 | 0.032 | 0.034 | 0.185 | 0.196 | 2.526 | 2.315 | 0.085 | 0.012 |
| 1976 | 6.049 | -0.485 | 117 | 26.157 | -0.365 | -0.012 | 0.019 | -0.067 | 0.101 | 2.128 | 1.320 | 0.071 | -0.008 |
| 1977 | 5.569 | -0.664 | 51 | 31.503 | 0.007 | 0.000 | 0.033 | 0.002 | 0.144 | 2.036 | 1.504 | 0.069 | 0.0002 |
| 1978 | 5.928 | -0.530 | 31 | 30.473 | 0.468 | 0.027 | 0.047 | 0.112 | 0.198 | 2.082 | 1.353 | 0.069 | 0.013 |
| 1979 | 6.274 | -0.401 | 89 | 27.453 | -0.220 | -0.006 | 0.020 | -0.037 | 0.118 | 2.256 | 1.281 | 0.074 | -0.005 |
| 1980 | 6.488 | -0.321 | 50 | 20.658 | -1.450 | -0.064 | 0.032 | -0.306 | 0.151 | 2.427 | 1.267 | 0.079 | -0.042 |
| 1981 | 8.972 | 0.605 | 32 | 20.407 | -1.641 | -0.042 | 0.032 | -0.263 | 0.200 | 7.985 | 3.080 | 0.200 | -0.065 |
| 1982 | 6.812 | -0.200 | 57 | 20.658 | -1.319 | -0.051 | 0.031 | -0.259 | 0.160 | 2.779 | 1.285 | 0.093 | -0.035 |
| 1983 | 6.755 | -0.221 | 40 | 25.602 | -0.027 | -0.002 | 0.056 | -0.008 | 0.202 | 2.709 | 1.279 | 0.086 | -0.002 |
| 1984 | 5.263 | -0.778 | 6 | 29.806 | 1.875 | 0.095 | 0.090 | 0.422 | 0.402 | 2.105 | 1.707 | 0.071 | 0.042 |
| 1985 | 7.201 | -0.055 | 39 | 24.811 | -2.552 | -0.080 | 0.033 | -0.451 | 0.188 | 3.350 | 1.374 | 0.108 | -0.100 |
| 1986 | 5.324 | -0.755 | 57 | 32.560 | 0.268 | 0.033 | 0.071 | 0.094 | 0.200 | 2.083 | 1.660 | 0.071 | 0.023 |
| 1987 | 6.764 | -0.218 | 73 | 24.027 | -1.216 | -0.071 | 0.039 | -0.295 | 0.160 | 2.720 | 1.280 | 0.091 | -0.055 |
| 1988 | 7.564 | 0.080 | 7 | 26.755 | -3.634 | -0.185 | 0.083 | -0.820 | 0.368 | 4.028 | 1.534 | 0.126 | -0.056 |
| 1989 | 7.563 | 0.080 | 31 | 22.717 | -1.541 | -0.042 | 0.036 | -0.253 | 0.217 | 4.026 | 1.533 | 0.126 | -0.044 |
| 1990 | 8.140 | 0.295 | 67 | 18.469 | -3.430 | -0.075 | 0.023 | -0.506 | 0.153 | 5.391 | 1.977 | 0.149 | -0.123 |
| 1991 | 8.727 | 0.514 | 34 | 21.072 | -0.635 | -0.014 | 0.031 | -0.093 | 0.214 | 7.145 | 2.698 | 0.185 | -0.019 |
| 1992 | 6.799 | -0.205 | 35 | 17.175 | -0.955 | -0.030 | 0.032 | -0.170 | 0.178 | 2.763 | 1.284 | 0.092 | -0.024 |
| 1993 | 7.571 | 0.083 | 20 | 25.115 | -0.621 | -0.052 | 0.069 | -0.179 | 0.240 | 4.041 | 1.538 | 0.119 | -0.052 |
| 1994 | 6.801 | -0.204 | 24 | 22.860 | -2.382 | -0.110 | 0.044 | -0.512 | 0.204 | 2.766 | 1.284 | 0.093 | -0.080 |
| 1995 | 6.937 | -0.154 | 71 | 28.767 | -0.733 | -0.043 | 0.036 | -0.178 | 0.147 | 2.945 | 1.306 | 0.097 | -0.038 |
| 1996 | 5.945 | -0.524 | 24 | 27.511 | 0.241 | 0.012 | 0.049 | 0.053 | 0.222 | 2.087 | 1.348 | 0.070 | 0.007 |
| 1997 | 7.344 | -0.002 | 20 | 17.916 | -0.724 | -0.022 | 0.039 | -0.126 | 0.223 | 3.600 | 1.427 | 0.115 | -0.026 |
| 1998 | 8.294 | 0.352 | 23 | 20.526 | -4.926 | -0.163 | 0.045 | -0.895 | 0.246 | 5.815 | 2.139 | 0.158 | -0.255 |
| 1999 | 8.231 | 0.329 | 13 | 20.018 | -2.192 | -0.068 | 0.054 | -0.386 | 0.305 | 5.639 | 2.070 | 0.155 | -0.077 |
| 2000 | 7.415 | 0.025 | 80 | 17.597 | -1.707 | -0.072 | 0.026 | -0.352 | 0.128 | 3.733 | 1.459 | 0.118 | -0.088 |
| 2001 | 6.242 | -0.413 | 30 | 29.059 | -3.384 | -0.173 | 0.041 | -0.764 | 0.183 | 2.234 | 1.285 | 0.073 | -0.077 |
| 2002 | 7.727 | 0.141 | 87 | 21.390 | -0.442 | -0.013 | 0.021 | -0.077 | 0.121 | 4.378 | 1.634 | 0.127 | -0.019 |
| 2003 | 7.409 | 0.022 | 16 | 21.268 | -1.843 | -0.115 | 0.059 | -0.460 | 0.236 | 3.721 | 1.456 | 0.118 | -0.128 |
| 2004 | 8.142 | 0.296 | 95 | 21.411 | -0.310 | -0.022 | 0.027 | -0.082 | 0.101 | 5.398 | 1.980 | 0.150 | -0.041 |
| 2005 | 10.029 | 1.000 | 24 | 15.794 | -1.010 | -0.040 | 0.046 | -0.201 | 0.230 | 12.348 | 5.278 | 0.268 | -0.146 |
| 2006 | 6.940 | -0.153 | 45 | 24.459 | -1.615 | -0.081 | 0.042 | -0.363 | 0.187 | 2.949 | 1.306 | 0.098 | -0.084 |
| 2007 | 9.990 | 0.985 | 40 | 15.024 | -3.779 | -0.058 | 0.021 | -0.469 | 0.169 | 12.162 | 5.180 | 0.266 | -0.191 |
