# Supplementary material for: Speeding Up Microevolution: The Effects of Increasing Temperature on Selection and Genetic Variance in a Wild Bird Population
Source: PLoS Biol. 2011 Feb 1;9(2):e1000585. doi: 10.1371/journal.pbio.1000585 (PMC3051266; doi:10.1371/journal.pbio.1000585)
Supplement: Text S1 — Environmentally dependent strength of standardized laying date selection and the covariance between standardized laying date selection with additive genetic variance and heritability. (0.03 MB DOC) [file pbio.1000585.s003.doc]

**PLoS Biology. Husby, Visser & Kruuk, supplementary material.**

To allow comparison with other studies, we report here the outcome of the statistical analyses using standardized selection gradients (i.e β = cov(w, z_ld_)/Vz_ld_, where z_ld_ is the variance-standardized measure of laying date, i.e. z_ld_ = (ld_i_ – μ_ld_)/sd_ld_) which, because Vz_ld_ =1, are equivalent to the standardized selection differentials. Note that in all analyses in the main text we use the observed laying dates and thus the presented selection gradients and differentials are unstandardized.

*Environmental dependence of strength of selection*

Similar to when using unstandardized laying dates, we found, firstly, strong selection on standardized laying date, with early breeding birds having higher fitness than late breeding individuals (Table S1) with 29 out of the 35 estimates of annual standardized selection gradients being negative (Table S2). Secondly, the interaction between standardized laying date and standardized spring temperature was significantly negative (Table S1), indicating that with increasing spring temperatures the relationship (slope) between fitness and standardized laying date became more negative (i.e. slope steeper in warmer years). This result was confirmed by regressing the annual standardized selection gradients against temperature: there was a significant increase in the strength of the standardized selection gradients with increasing temperatures (b = -0.12, se = 0.04, t_33_ = -3.26, *P* = 0.003 using 1/se^2^ as weights).

*Association between strength of selection and additive genetic variance*

As when using unstandardized selection gradients we found that there was a negative, although non-significant, relationship between the strength of selection and additive genetic variance (b= -0.03, se = 0.02, t_33_= -1.53, *P* = 0.136, using 1/se^2^ as weights) which was marginally non-significant when excluding the extreme V_A_ outliers (b= -0.065, se = 0.033, t_33_= -1.98, *P* = 0.057, using 1/se^2^ as weights).

Similarly, there was a marginally non-significant relationship between the selection gradients and heritability (b= -1.94, se = 1.004, t_33_= -1.93, *P* = 0.062, using 1/se^2^ as weights) which was significant when excluding the extreme h2 outliers (b= -3.36, se = 1.42, t_33_= -2.36, *P* = 0.024, using 1/se^2^ as weights). Note that because there is selection for early breeding, selection gradients are negative, but there is a *positive* association between the *strength* of selection and heritability).

*Predicting the response to selection*

Ultimately we are interested in how the positive covariance observed here between strength of selection and expression of additive genetic variance (and heritability) influence the population’s ability to respond to selection. However, we would like to emphasize that predicting a response to selection in natural populations is not a trivial thing and has limited accuracy in situations in which there is environmental covariance of the trait and fitness or, for example, where other traits that are not included in the analysis influence the trait and fitness (for further explanation on this see Merilä et al 2001, Kruuk et al 2008). As a result, the Robertson-Price genetic covariance between a trait and fitness may be a better predictor of the cross-generation response to selection (e.g. Hadfield PRSB 2008, Morrisey et al 2010). However, in our study population there is little evidence of an environmental covariance between fitness and laying date (Gienapp et al. 2006) suggesting that our estimates of phenotypic selection accurately reflect also the underlying genetic covariance between laying date and fitness, thus giving a relatively accurate measure of the strength of selection.

With the above caveats in mind, we predicted the response to selection following traditional methodology using the Lande equation (R= V_A_*β, Lande 1979), but correcting for overlapping generations and the sex-limited expression of laying date. Following Gienapp et al. (2006) we therefore estimated the annual response to selection as:

R = VA *β * 0.5 *Prec_t+1_, where Prec_t+1_ is the proportion of adults being first year breeders in year t+1 and is used as a proxy to correct for overlapping generation time (see Gienapp et al. 2006). The factor 0.5 is used to correct for the fact that laying date is a sex specific trait. To get the predicted response across all years we then calculated the cumulative response.

We strongly emphasize however that our predictions should be interpreted with caution, given the extensive evidence that the breeder’s equation is often too simplistic a representation of evolutionary dynamics in natural systems to generate accurate predictions (Merila et al. 2001; Kruuk et al 2008).

**References:**

Gienapp P, Postma EP, Visser ME (2006) Why breeding time has not responded to selection for earlier breeding in a songbird population. Evolution 60: 2381-2388.

Hadfield JD (2008) Estimating evolutionary parameters when viability selection is operating. Proceedings of the Royal Society B-Biological Sciences 275: 723-735.

Kruuk LEB, Slate J, Wilson AJ (2008) New Answers for Old Questions: The Evolutionary Quantitative Genetics of Wild Animal Populations. Annual Review of Ecology Evolution and Systematics 39: 525-548

Lande R (1979) Quantitative genetic analysis of multivariate evolution, applied to brain body size allometry. Evolution 33: 402-416.

Merilä J, Sheldon BC, Kruuk LEB (2001) Explaining stasis: microevolutionary studies in natural populations. Genetica 112: 199-222.

Morrisey MD, Kruuk LEB, Wilson AJ (2010) The danger of applying the breeder’s equation in observational studies of natural populations. Journal of Evolutionary Biology 23: 2277-2288.
